# Structural basis for T cell recognition of cancer neoantigens and implications for predicting neoepitope immunogenicity

**DOI:** 10.3389/fimmu.2023.1303304

**Published:** 2023-11-17

**Authors:** Roy A. Mariuzza, Daichao Wu, Brian G. Pierce

**Affiliations:** ^1^W.M. Keck Laboratory for Structural Biology, University of Maryland Institute for Bioscience and Biotechnology Research, Rockville, MD, United States; ^2^Department of Cell Biology and Molecular Genetics, University of Maryland, College Park, MD, United States; ^3^Laboratory of Structural Immunology, Department of Hepatopancreatobiliary Surgery, The First Affiliated Hospital, Hengyang Medical School, University of South China, Hengyang, Hunan, China

**Keywords:** cancer neoantigen, T cell, TCR, MHC, immunotherapy, immunogenicity, structure

## Abstract

Adoptive cell therapy (ACT) with tumor-specific T cells has been shown to mediate durable cancer regression. Tumor-specific T cells are also the basis of other therapies, notably cancer vaccines. The main target of tumor-specific T cells are neoantigens resulting from mutations in self-antigens over the course of malignant transformation. The detection of neoantigens presents a major challenge to T cells because of their high structural similarity to self-antigens, and the need to avoid autoimmunity. How different a neoantigen must be from its wild-type parent for it to induce a T cell response is poorly understood. Here we review recent structural and biophysical studies of T cell receptor (TCR) recognition of shared cancer neoantigens derived from oncogenes, including p53^R175H^, KRAS^G12D^, KRAS^G12V^, HHAT^p8F^, and PIK3CA^H1047L^. These studies have revealed that, in some cases, the oncogenic mutation improves antigen presentation by strengthening peptide–MHC binding. In other cases, the mutation is detected by direct interactions with TCR, or by energetically driven or other indirect strategies not requiring direct TCR contacts with the mutation. We also review antibodies designed to recognize peptide–MHC on cell surfaces (TCR-mimic antibodies) as an alternative to TCRs for targeting cancer neoantigens. Finally, we review recent computational advances in this area, including efforts to predict neoepitope immunogenicity and how these efforts may be advanced by structural information on peptide–MHC binding and peptide–MHC recognition by TCRs.

## Introduction

Adaptive cell therapy (ACT) with tumor-specific T cells has been demonstrated to mediate durable cancer regression in patients with metastatic melanoma, breast, cervix, colon, and bile duct cancers (1–6). The therapeutic effect of these *ex vivo*-expanded tumor-infiltrating lymphocytes (TILs) is principally mediated by CD8^+^ cytotoxic T cells (7), with an additional contribution from CD4^+^ T cell (5). The principal target of tumor-specific T cells are neoantigens resulting from non-synonymous somatic mutations in self-antigens during malignant transformation (6, 8). The identification of neoantigens in individual patients, and of the T cells that recognize them, has been greatly accelerated by recent technical advances in high-throughput T cell-based assays and mass spectrometry (9).

A daunting challenge in the development of broadly useful neoantigen-based ACT is the unique neoantigen repertoire of individual cancer patients (private neoantigens) (8). There exist few common neoantigens among patients, even for patients with similar cancers, that can be targeted therapeutically (public neoantigens). For instance, an analysis of patients with gastrointestinal cancers found that nearly all (99%) of neoantigenic determinants (neoepitopes) targeted by neoantigen-reactive TILs were private (10). Nevertheless, a few public cancer neoantigens have been discovered (11–16). Of particular interest are neoantigens derived from oncogenes such as *TP53* and *KRAS* that bear driver mutations. This is because driver mutations are tumor-specific, essential for cancer cell fitness and proliferation, and likely to be present in every cell within the cancer (17).

The detection of neoantigens represents a major challenge to T cells because of their high similarity to wild-type self-peptides. Exactly how different a neoantigen must be from its wild-type parent for it to overcome self-tolerance and induce a T cell response is poorly understood. With the goal of understanding T cell recognition of cancer neoantigens at the molecular level, a number of crystal structures have been recently reported of TCRs bound to various cancer neoantigens and MHC class I or class II molecules (**Table 1**) (18–27). These neoantigens include p53^R175H^ (20, 21), KRAS^G12D^ (22, 23, 27), KRAS^G12V^ (24), HHAT^p8F^ (25), PIK3CA^H1047L^ (26), and TPI^T28I^ (18, 19). Structures have also been determined for TCRs bound to epitopes from the tumor-associated antigens NY-ESO-1, MART-1, MAGEA4, and Melan A bound to HLA-A2 (28–31). However, it is important to note these are not neoantigens but rather unmutated self-antigens that are selectively expressed or overexpressed in certain types of cancer.

**Table 1:** Structures of TCR–pMHC complexes involving cancer neoantigens.

TCR–pMHC complex	PDB code (reference)	Neoepitope	Affinity wild-type	Affinity mutant	Basis for neoepitope immunogenicity
E8–TPI^T28I^–HLA-DR1	2IAM (18)	GELIG**I**LNAAKVPAD	UD	>300 μM	Direct TCR contacts with mutation
G4–TPI^T28I^–HLA-DR1	4E41 (19)	GELIG**I**LNAAKVPAD	UD	>300 μM	Direct TCR contacts with mutation
12-6–p53^R175H^–HLA-A*02:01	6VRM (20)	HMTEVVR**H**C	UD	1.1 μM	Direct TCR contacts with mutation
38-10–p53^R175H^–HLA-A*02:01	6VRN (20)	HMTEVVR**H**C	UD	39.9 μM	Direct TCR contacts with mutation
1a2–p53^R175H^–HLA-A*02:01	6VQO (20)	HMTEVVR**H**C	UD	16.2 μM	Direct TCR contacts with mutation
6-11–p53^R175H^–HLA-A*02:01	7RM4 (21)	HMTEVVR**H**C	214 μM	3.5 μM	No direct TCR contacts with mutation Reduced energetic cost of desolvating mutation during TCR engagement
9a–KRAS^G12D^–HLA-C*08:02	6ULN (22)	GA**D**GVGKSA	NA	16 nM	Stabilization of pMHC ligand by anchor residue mutation
9d–KRAS^G12D^–HLA-C*08:02	6ULR (22)	GA**D**GVGKSA	NA	125 nM	Stabilization of pMHC ligand by anchor residue mutation
10–KRAS^G12D^–HLA-C*08:02	6UON (22)	GA**D**GVGKSAL	NA	6.7 μM	Stabilization of pMHC ligand by anchor residue mutation
JDI–KRAS^G12D^–HLA-C*11:01	7PB2 (23)	VVVGA**D**GVGK	UD	63 μM	No direct TCR contacts with mutation Formation of new electrostatic interactions of mutant peptide with TCR
1-2C–KRAS^G12V^–HLA-A*11*01	8I5C (24)	VVGA**V**GVGK	131 μM	14 μM	Direct TCR contacts with mutation
3-2E–KRAS^G12V^–HLA-A*11*01	8I5D (24)	VVGA**V**GVGK	42 μM	28 μM	Direct TCR contacts with mutation
302TIL–HHAT^p8F^–HLA-A*02:06	6UK4 (25)	KQWLVWL**F**L	200 μM	9 μM	Conformational pre-organization of pMHC ligand by mutation
3–PIK3CA^H1047L^–HLA-A*03:01	7RRG (26)	A**L**HGGWTTK	ND	200 μM	Stabilization of pMHC ligand by anchor residue mutation
4–PIK3CA^H1047L^–HLA-A*03:01	7L1D (26)	A**L**HGGWTTK	ND	49 μM	Stabilization of pMHC ligand by anchor residue mutation

UD, undetectable; NA, not applicable; ND, not determined.

Collectively, structural studies of mutated self-antigens have provided insights into the multiple mechanisms TCRs employ to detect cancer neoantigens and into how mutations confer immunogenicity to normally cryptic self-peptides. In some cases, the mutation strengthens peptide–MHC binding, improving the presentation of neoepitopes against which the immune system is not tolerant. In other cases, the mutation does not affect peptide–MHC binding or antigen presentation, yet generates a peptide that is sufficiently different physically from its wild-type parent to be immunogenic. Detection of such peptides by T cells may occur via direct interactions between TCR and the oncogenic mutation, or may involve energetically driven or other indirect strategies not requiring direct contacts.

Here we review structural and biophysical studies of TCR recognition of cancer neoantigens with a focus on how T cells distinguish mutant from wild-type epitopes and how neoepitope-specific TCRs may be employed for ACT. We also review work on designing antibodies that mimic TCR recognition of pMHC on cell surfaces as an alternative approach to immunotherapeutic targeting of cancer neoantigens. Finally, we review recent computational efforts to predict neoepitope immunogenicity and how some of these efforts have utilized structural information on peptide binding to MHC and pMHC recognition by TCRs.

### TCR recognition of TPI^T28I^–HLA-DR1

The first structural studies of TCR recognition of a cancer neoantigen involved two tumor-specific TCRs (E8 and G4) isolated from TILs of a melanoma patient in complex with the MHC class II molecule HLA-DR1 and a mutated self-peptide derived from the glycolytic enzyme triosephosphate isomerase (TPI) (**Table 1**) (18, 19). A natural mutation in TPI resulted in replacement of an isoleucine residue for threonine in a neoepitope corresponding to residues 23–37 of TPI^T28I^ (GELIG**I**LNAAKVPAD; mutant amino acid in bold) (32). This substitution resulted in >100,000-fold improved recognition of mutant TPI^T28I^ relative to wild-type TPI, thereby revealing this neoepitope to T cells (18, 32). However, the TPI^T28I^ neoantigen, unlike neoantigens such as p53^R175H^ and KRAS^G12V^ that are derived from mutant oncogenes, was only expressed in a single melanoma patient. This limitation precludes wide use of TPI^T28I^-specific or similar TCRs in ACT.

Surprisingly, TCRs E8 and G4 displayed very low affinities for TPI^T28I^–HLA-DR1, as measured by surface plasmon resonance (SPR), with dissociation constants (*K*_D_s) exceeding 300 μM (**Table 1**) (18). However, as measured by two-dimensional (2D) mechanical-based adhesion assays, the 2D affinity of TCR E8 for TPI^T28I^–HLA-DR1 was comparable to other TCR–pMHC class II interactions (33). Importantly, 2D measurements are made *in situ* at cell–cell junctions, whereas three-dimensional (3D) measurements from SPR use soluble proteins isolated from their cellular contexts. As such, 2D parameters correspond to biology much better than their 3D counterparts (34–37). No TCR binding to wild-type TPI–HLA-DR1 was detected, in agreement with functional assays showing greatly diminished T cell activation by the wild-type peptide (18).

**Figure 1:**
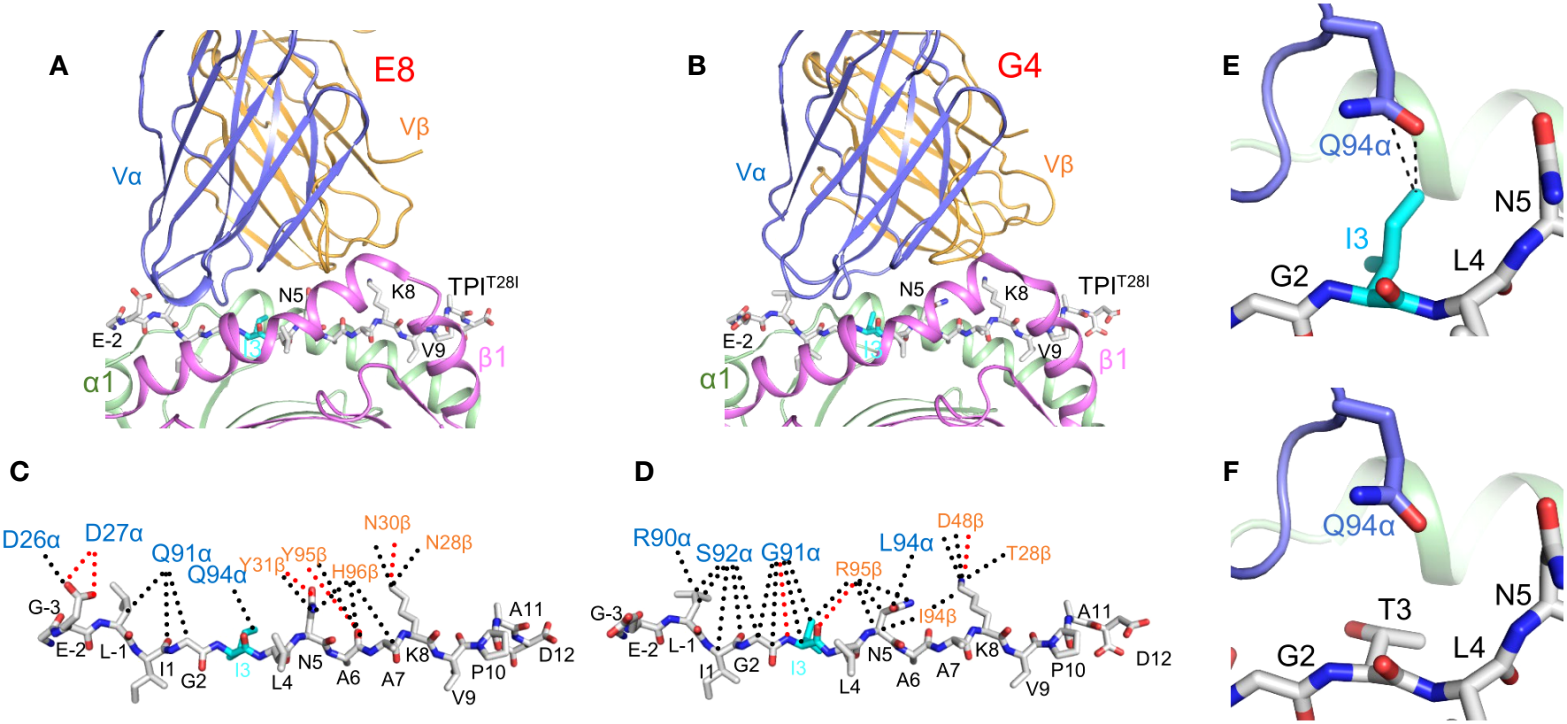
TCR recognition of the HLA-DR1-restricted TPI^T28I^ cancer neoantigen. **(A)** Interactin of TCR E8 with TPI^T28I^ and HLA-DR1 (ribbon diagram) (PDB accession code 2IAM) (18). TCR α chain, blue; TCR β chain, gold; MHC α chain, green; MHC β chain, magenta. The peptide is in ball-and-stick format, with carbon atoms in gray, nitrogen atoms in blue, and oxygen atoms in red. **(B)** Interaction of TCR G4 with TPI^T28I^ and HLA-DR1 (4E42) (19). **(C)** Interactions between TCR E8 and TPI^T28I^. Peptide residues are identified by a one-letter amino acid designation, followed by position number. Hydrogen bonds are red dotted lines and van der Waals contacts are black dotted lines. For clarity, not all van der Waals contacts are shown. The mutant P3 Ile residue is cyan. **(D)** Interactions between TCR G4 and TPI^T28I^. **(E)** Van der Waals contacts (black dotted lines) between TCR E8 and mutant P3 Ile residue. **(F)** Absence of contacts between E8 and wild-type P3 Thr residue (2IAN) (18).

Replacement of isoleucine by threonine at position P3 of TPI^T28I^ did not alter peptide affinity for HLA-DR1 or affect the conformation of the peptide in the MHC binding groove (38). Rather, structural changes in the TCR–pMHC interface are primarily responsible for improved T cell recognition of TPI^T28I^. TCRs E8 and G4 utilize the same Vα region (TRAV13-1) but have different CDR3α sequences and different Vβ regions (TRBV6-6 and TRBV5-8, respectively). Crystal structures of E8 and G4 bound to TPI^T28I^–HLA-DR1 showed that both TCRs dock on pMHC in the canonical diagonal orientation, with the Vα domain over the β1 helix of HLA-DR1 and the Vβ domain over the α1 helix (**Figures 1A, B**) (18, 19). However, E8 and G4 recognize TPI^T28I^ in markedly different ways. In the E8–TPI^T28I^–HLA-DR1 complex, the CDR1 and CDR3 loops of both Vα and Vβ make similar contributions to contacts with TPI^T28I^ (**Figure 1C**). By contrast, in the G4–TPI^T28I^–HLA-DR1 complex, peptide recognition is mediated almost completely by CDR3α and CDR3β (**Figure 1D**).

In the E8–TPI^T28I^–HLA-DR1 complex, CDR3α and CDR3β form a dome-shaped pocket that accommodates two TPI^T28I^ residues, P3 Ile (the mutant amino acid) and P5 Asn. The δ1 methyl group of P3 Ile projects from the TPI^T28I^–HLA-DR1 surface towards CDR3α Gln94, with which it forms multiple van der Waals contacts (**Figure 1E**). Replacement of P3 Ile by threonine (the wild-type amino acid) results in loss of these contacts, a reduction in shape complementarity, and a decrease in buried surface at the mutation site, where P3 Ile occupies a mainly hydrophobic pocket on TCR E8 (**Figure 1F**) (18). In the G4–TPI^T28I^–HLA-DR1 complex, the main chain of P3 Ile makes three hydrogen bonds with CDR3α Gly91 and CDR3β Arg (19). In the structure of wild-type TPI–HLA-DR1 (38), P3 Thr, unlike P3 Ile, is completely buried against the HLA-DR1 α chain. This renders P3 Thr inaccessible to TCR G4, which results in a strong preference for isoleucine over threonine at P3 in T cell activation assays (18).

### TCR recognition of p53^R175H^–HLA-A2

*TP53* (tumor protein 53) was the first tumor suppressor gene to be discovered (39). It is inactivated in the large majority of human cancers (40) and is the most frequently mutated gene across all cancers (41). Driver mutations in *TP53* cause most of the key features of cancer cells, notably genomic instability, proliferation, and metastasis (42, 43). A high percentage of *TP53* mutations are located at positions R175, G245, R248, R273, and R282. These mutations cluster in the central DNA-binding domain of p53 and affect DNA binding (41). They are attractive targets for immunotherapy because they are associated with tumor progression and confer a growth advantage to cancer cells.

The immunogenicity of p53 mutations was demonstrated by the detection in cancer patients of T cell responses against several shared p53 neoantigens, mainly R175H and R248W (16, 44). The R175H driver mutation is the most frequent mutation in *TP53*. It is also the most common mutation in any tumor suppressor gene (45). Several TCRs (12-6, 38-10, 1a2, and 6-11) have been isolated from patients with epithelial cancers that recognize a neoepitope corresponding to residues 168–176 of p53^R175H^ (HMTEVVR**H**C; mutant amino acid in bold) (16, 44). These TCRs are restricted by HLA-A*02:01, which is the most common MHC class I allele in the U.S. and Chinese populations (46). In a clinical trial, a breast cancer patient infused with autologous peripheral blood lymphocytes transduced with an HLA-A*02-restricted TCR (6–11) specific for p53^R175H^ experienced ~55% tumor regression that lasted 6 months (47).

As measured by SPR, TCRs 12-6, 38-10, and 1a2 exhibited exquisite specificity for p53R175H–HLA-A2, with *K*_D_s ranging from 1 μM to 40 μM and no detectable binding to wild-type p53–HLA-A2 (**Table 1**). These results agree with functional assays showing that T cells transduced with these TCRs could be triggered by very low (subnanomolar) concentrations of mutant p53^R175H^ peptide but not by wild-type p53 peptide (16, 44). By contrast, TCR 6-11 was not as highly specific for p53^R175H^–HLA-A2 as TCRs 12-6, 38-11 or 1a2, since its affinity for p53^R175H^–HLA-A2 (*K*_D_ = 3.5 μM) was only ~60-fold higher than for p53–HLA-A2 (*K*_D_ = 214 μM) (**Table 1**) (21). TCR affinities for the p53^R175H^ neoantigen are comparable to those of TCRs specific for viral or other foreign antigens (*K*_D_ = 1–50 μM) but substantially higher than the affinities of autoimmune TCRs specific for unmutated self-antigens (*K*_D_ > 100 μM) (48). These affinity characteristics also apply to MHC class I-restricted TCRs that recognize other neoantigens (**Table 1**).

**Figure 2:**
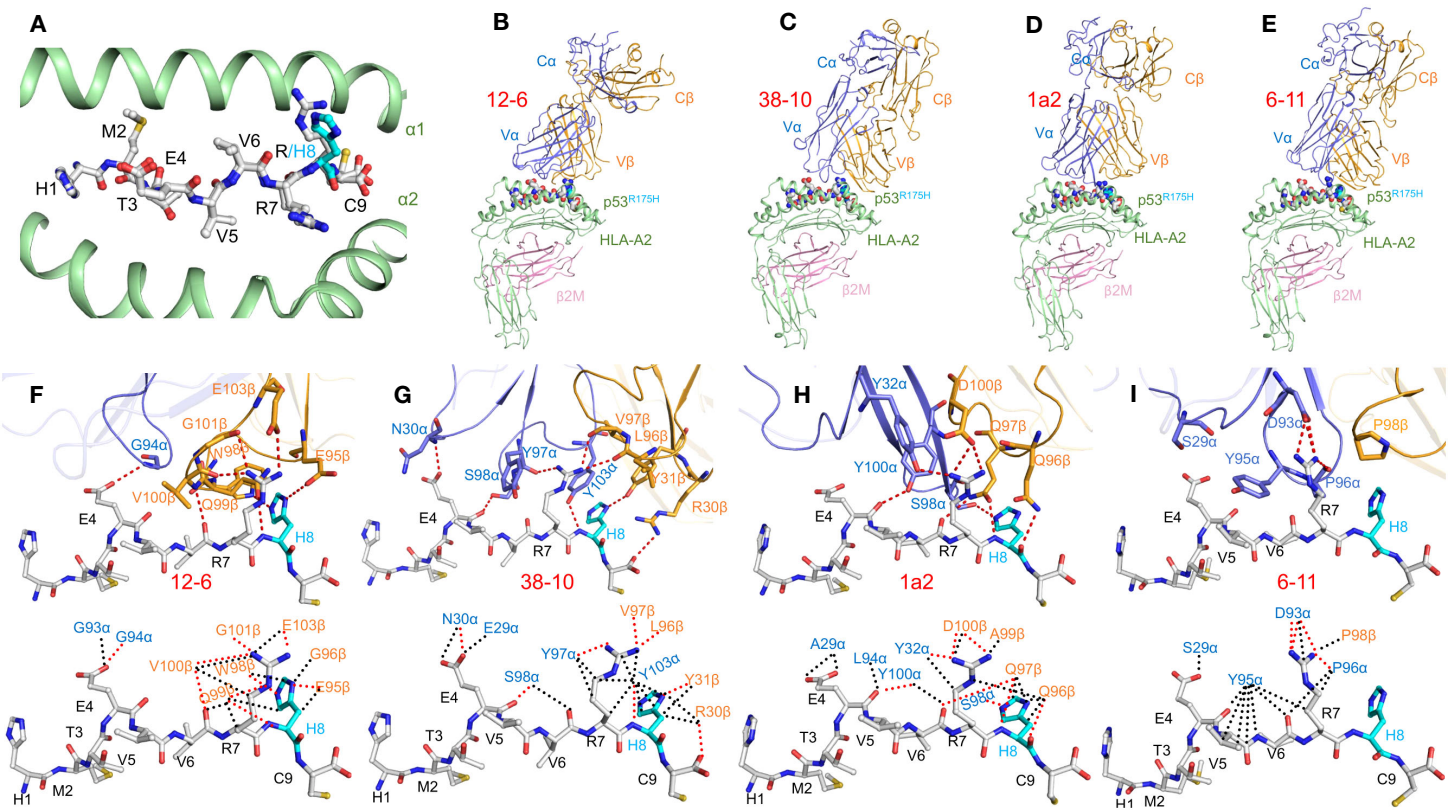
TCR recognition of the HLA-A2-restricted p53^R175H^ neoepitope. **(A)** Conformation of wild-type and mutant p53 peptides bound to HLA-A2. Top view of the superposed p53–HLA-A2 and p53^R175H^–HLA-A2 complexes (6VR1 and 6VR5) (20). Carbon atoms of wild-type and mutant p53 peptides are gray; nitrogen atoms are blue; oxygen atoms are red; sulfur atoms are orange. The mutant P8 His residue is cyan. HLA-A2 is green. **(B–E)** Side view of the 12-6–p53^R175H^–HLA-A2, 38-10–p53^R175H^–HLA-A2, 1a2–p53^R175H^–HLA-A2, and 6-11–p53^R175H^–HLA-A2 complexes (6VRM, 6VRN, 6VQO, and 7RM4) (20, 21). TCR α chain, blue; TCR β chain, gold; HLA-A2 heavy chain, green; β_2_-microglobulin (β_2_m), pink. **(F–I)** (upper panels) Interactions between TCRs 12-6, 38-10, 1a2, and 6-11 and the p53^R175H^ peptide. The side chains of contacting residues are shown in stick representation. The mutant P8 His residue is cyan. (lower panels) Comparison of interactions between 12-6, 38-10, 1a2, and 6-11 and the p53^R175H^ peptide. Hydrogen bonds are red dotted lines and van der Waals contacts are black dotted lines.

Structures of the wild-type p53–HLA-A2 and mutant p53^R175H^–HLA-A2 complexes were determined in order to understand how the conservative arginine-to-histidine mutation in p53, which replaces one positively charged residue by another, renders p53^R175H^ immunogenic (**Figure 2A**) (20). In both complexes, the side chains of anchor residues P2 Met and P9 Cys are situated in the peptide-binding groove of HLA-A2, while the side chains of P1 His, P4 Glu, P7 Arg, and P8 Arg/His extend up from the groove. Comparison of the p53–HLA-A2 and p53^R175H^–HLA-A2 complexes showed that structural differences that revealed the p53^R175H^ peptide to T cells (16, 44) are confined to the mutation site at P8 (**Figure 2A**) (20).

In order to understand how TCRs 12-6, 38-10, 1a2, and 6-11 discriminate between wild-type and mutant p53 epitopes, structures were determined of their corresponding complexes with p53^R175H^–HLA-A2 (20, 21) (**Figures 2B–E**). These TCRs utilize unrelated Vα and Vβ gene segments. TCRs 12-6, 38-10, and 1a2, but not 6-11, are displaced towards the C-terminus of the p53^R175H^ peptide. Importantly, this is the site of the driver mutation at P8 (**Figures 2F–I**). As a consequence, ~80% of contacts between TCRs 12-6, 38-10, and 1a2 and the p53^R175H^ peptide involves C-terminal residues P7 Arg and P8 His, in contrast to most TCRs, including 6-11, which typically target the central portion of peptides (P4–P6) (**Figure 2I**) (21). In each case, the imidazole ring of P8 His is tightly sandwiched between the HLA-A2 α1 helix and the TCR CDR3 loops (**Figures 2F–H**). TCRs 12-6, 38-10, and 1a2 discriminate between mutant and wild-type p53 by focusing on the R175H mutation at P8 and minimizing interactions with the central and N-terminal portions of p53^R175H^, which are structurally identical in the wild-type peptide (20). The dramatic loss of affinity for wild-type p53 is mainly due to disruption side chain–side chain hydrogen bond interactions involving P8 His upon replacing this residue by arginine.

In sharp contrast to TCRs 12-6, 38-10, and 1a2, TCR 6-11 makes no direct contacts with the R175H mutation (**Figure 2I**), but is nevertheless able to distinguish mutant from wild-type p53 (21). Since the peptide residues that do contact 6-11 are highly superimposable in the structures of mutant p53^R175H^–HLA-A2 and unbound wild-type p53–HLA-A2 (**Figure 2A**), the mechanism underlying discrimination is not obvious. However, structure-based *in silico* mutagenesis revealed that the 60-fold loss in 6-11 binding affinity for wild-type p53 compared to p53^R175H^ is attributable to the greater energetic cost of desolvating R175 in the wild-type p53 peptide than H175 in the mutant during complex formation (21). This indirect strategy for neoantigen recognition by 6-11 is fundamentally different from the direct strategies used by most other TCRs and emphasizes the multiple mechanisms T cells may employ to recognize tumor but not normal cells.

### TCR recognition of KRAS^G12D^–HLA-C*08:02

Mutations in the *KRAS* oncogene occur in ~15% of all cancers, with especially high frequencies in colorectal and pancreatic cancers (49). *KRAS* encodes a small GTPase that alternates between an inactive GDP-bound state and an active GTP-bound state which regulates cell survival, growth, and differentiation (50). A hotspot for oncogenic mutations in the KRAS protein is glycine at position 12, with G12D, G12V, and G12C the most common mutations (51). These driver mutations impair GTPase hydrolytic activity and lock KRAS in the active state, leading to constitutive oncogenic signaling (52). Crystal structures have been determined of TCRs bound to KRAS^G12D^ (22, 23) and KRAS^G12V^ (24) neoepitopes presented by HLA-C*08:02 and HLA-A*11:01 MHC class I molecules, respectively (**Table 1**).

In a landmark clinical study of ACT, a patient with metastatic colorectal cancer was treated with four different CD8^+^ T cell clones that targeted a KRAS^G12D^ neoepitope in the context of HLA-C*08:02 (2). All metastases that retained HLA-C*08:02 expression underwent regression. TCRs from these four clones (9a, 9b, 9c, and 9d) recognized a nonamer of KRAS^G12D^ (GA**D**GVGKSA) bound to HLA-C*08:02 (2), while a fifth TCR (TCR10) was decamer-specific (GA**D**GVGKSAL) (27). In marked contrast to wild-type TPI and p53 peptides, which bound tightly to MHC (18, 20), wild-type nonamer and decamer KRAS peptides did not, as indicated by the failure of these peptides, unlike their mutant counterparts, to stabilize HLA-C expression on TAP-deficient cells (22). This result suggested that TCR tumor specificity arose from preferential KRAS^G12D^ presentation by HLA-C rather than differential TCR recognition of KRAS^G12D^ over wild-type KRAS.

**Figure 3:**
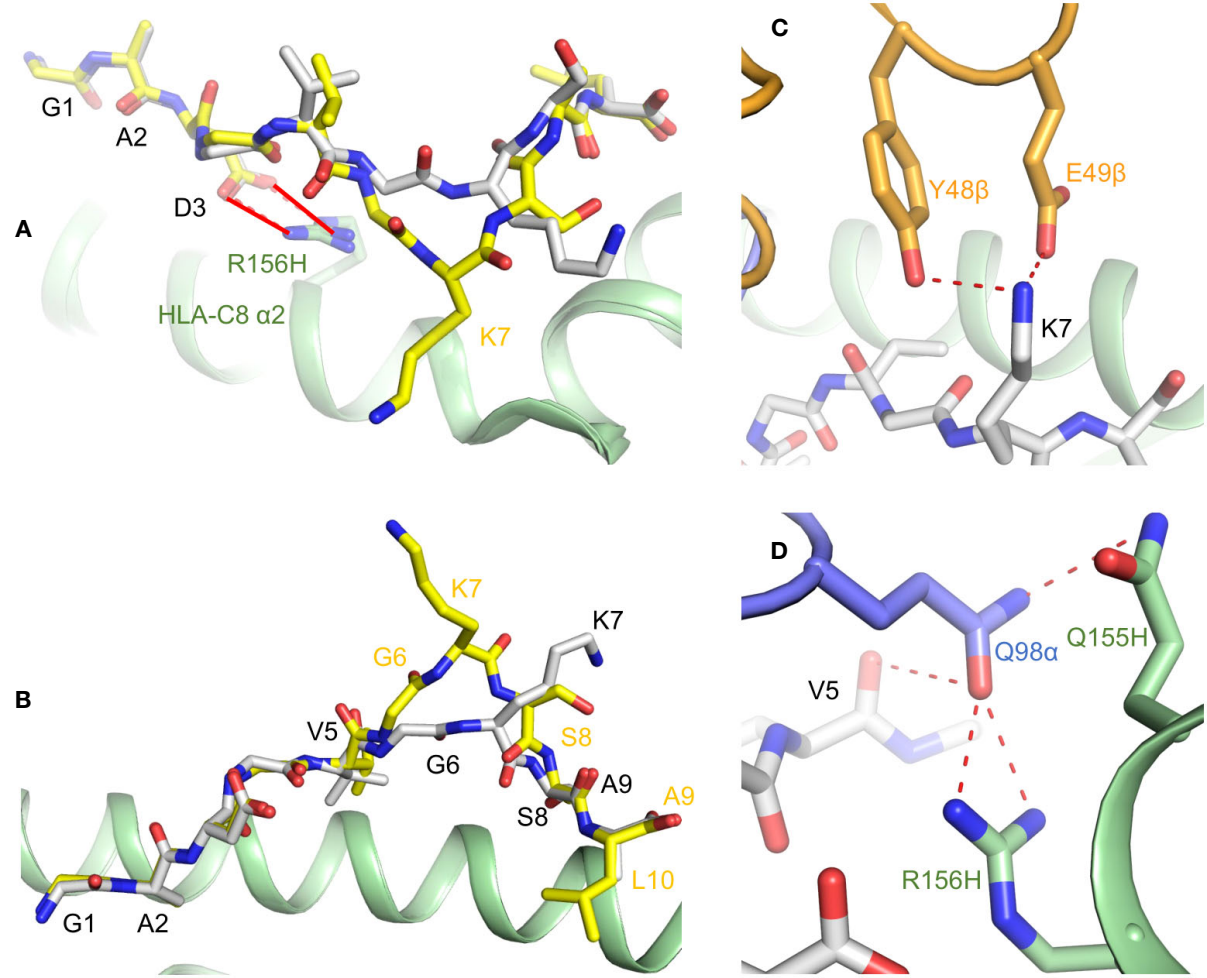
Presentation of KRAS^G12D^ neoepitopes by HLA-C*08:02. **(A)** KRAS^G12D^ nonamer and decamer peptides (gray and yellow, respectively) form a salt bridge (red solid lines) with Arg156 of the HLA-C α2 helix (green) through the mutant P3 Asp anchor residue (6ULI and 6ULK) (22). Wild-type P3 Gly cannot make this salt bridge. **(B)** Structures KRAS^G12D^ nonamer and decamer peptides bound to HLA-C*08:02. **(C)** Interactions of P7 Lys of KRAS^G12D^ nonamer with CDR2β Tyr48 and Glu49 of TCR9a (orange) (6ULN) (22). **(D)** Interactions of CDR3α Gln98 of TCR9a (blue) with P5 Val of KRAS^G12D^ nonamer and Gln155 and Arg156 of the HLA-C α2 helix.

Crystal structures of HLA-C*08:02 in complex with KRAS^G12D^ nonamer and decamer peptides revealed that, in both cases, P3 Asp (the mutant residue) makes a salt bridge with Arg156 on the HLA-C α2 helix (**Figure 3A**) (22). This salt bridge cannot form with P3 Gly, which probably explains the instability of wild-type KRAS–HLA-C complexes and their inability to activate T cells. The nonamer and decamer KRAS^G12D^ peptides are anchored to HLA-C via similar interactions at the N- and C-termini, but adopt different conformations at the center between P5 Val and P8 Ser due to a bulge at P7 Lys in the decamer (**Figure 3B**).

Structures of TCR9a and TCR9d (both Vα4/Vβ5) bound to KRAS^G12D^ nonamer and HLA-C showed that, in both complexes, P7 Lys forms a hydrogen bond with CDR2β Tyr48 and a salt bridge with CDR2β Glu49 (**Figure 3C**). In addition, CDR3α Gln98 makes hydrogen bonds with the carbonyl group of P5 Val and with Gln155 and Arg156 of the HLA-C α2 helix (**Figure 3D**). The different conformations of nonamer and decamer KRAS^G12D^ peptides (**Figure 3B**) result in distinct interactions with nonamer- and decamer-specific TCRs. For example, while both nonamer-specific TCR9a and decamer-specific TCR10 form salt bridge interactions with P7 Lys, TCR10 uses CDR3β Asp95 rather than CDR2β Glu49.

As measured by SPR, the affinities (*K*_D_s) of KRAS^G12D^-specific TCRs ranged from a typical 6 μM (TCR10) to an exceptionally high 16 nM (TCR9a) (**Table 1**) (22). In the case of treating a colorectal cancer patient with *ex vivo* expanded TILs specific for KRAS^G12D^ presented by HLA-C*08:02, the transferred T cells expressed four TCRs, TCR9a, 9b, 9c, and 10 (2). Surprisingly, T cells bearing TCR9a, the highest-affinity receptor, were undetectable 40 days post-transfer. Indeed, an inverse correlation was observed between TCR affinity and *in vivo* persistence, with T cells expressing TCR10, the lowest-affinity receptor, maintained in the periphery the longest (9 months post-transfer) (2). A possible explanation for this counterintuitive result is that higher-affinity T cells engage their cognate antigen more effectively, leading to increased activation-induced cell death (AICD), in which activation through the TCR results in apoptosis rather than proliferation (17, 53). This clinical study, while limited to a single patient, suggests that TCRs with affinities in the low micromolar range may be most efficacious for ACT.

### TCR recognition of KRAS^G12D^–HLA-A*11:01

Poole et al. (23) isolated a TCR (JDI) from the peripheral blood mononuclear cells (PBMC) of a healthy donor that recognizes a KRAS^G12D^ decamer peptide (VVVGA**D**GVGK) presented by HLA-A*11:01. This peptide partially overlaps, but is distinct from, the KRAS^G12D^ peptides presented by HLA-C*08:02 discussed above. TCR JDI bound mutant KRAS^G12D^–HLA-A*11 with *K*_D_ = 63 μM, with no measurable affinity for ubiquitously expressed wild-type KRAS–HLA-A*11 (**Table 1**), in agreement with functional assays using T cells transduced with JDI (23). With the aim of maximizing the anti-tumor activity of TCR JDI for possible immunotherapeutic applications, phage display was used to engineer a variant (JDIa41b1) with a 10^6^-fold affinity improvement (*K*_D_ = 0.7 pM) over the parental TCR that retained the ability to distinguish KRAS^G12D^ from wild-type KRAS, although JDIa41b1 did acquire measurable affinity for KRAS–HLA-A*11 (*K*_D_ = 3 μM).

A major concern with engineered high-affinity TCRs is the risk of cross-reactivity (54), which may result in adverse clinical events (55). In a striking case, an affinity-enhanced TCR targeting the MAGE-A3 melanoma antigen unexpectedly cross-reacted with an epitope from the muscle protein titin, resulting in cardiovascular toxicity and death in two patients who received cells transduced with the modified TCR (56). To address this concern for affinity-enhanced JDIa41b1, the TCR was panned against a phage-displayed peptide–HLA-A*11 library encoding >10^6^ variants to generate a peptide specificity profile (23). This profile was then used to identify *bona fide* self-peptides that might act as structural mimics of the neoepitope. However, none of these self-peptides were recognized by TCR JDIa41b1, easing concerns about potentially deleterious cross-reactivity. In addition, the affinity-enhanced TCR, fused to an anti-CD3 single-chain Fv fragment, mediated selective killing of cancer cells expressing KRAS^G12D^ (23).

**Figure 4:**
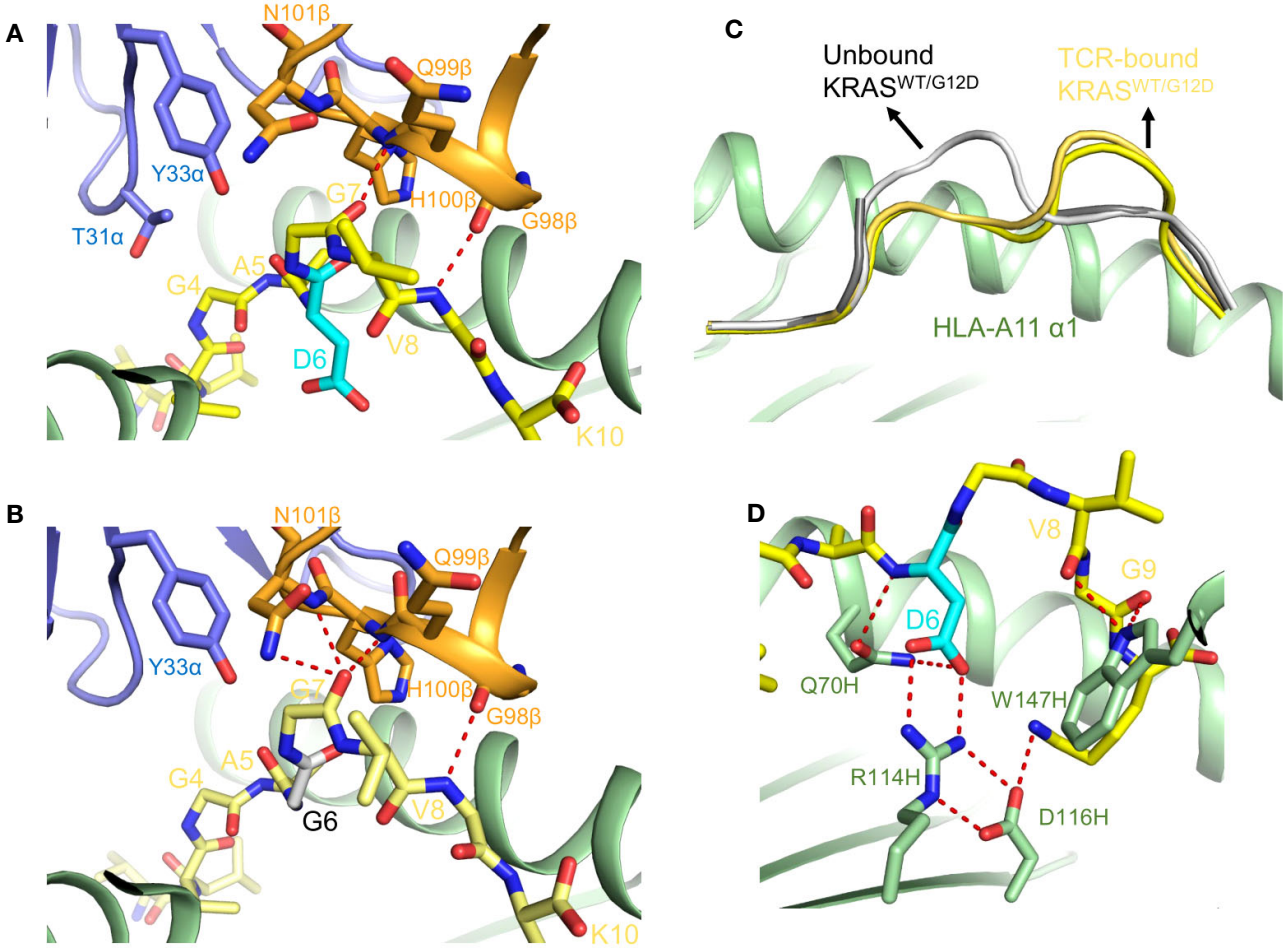
TCR recognition of the HLA-A*11:01-restricted KRAS^G12D^ neoepitope. **(A)** Interactions between TCR JDI and the KRAS^G12D^ peptide (7PB2) (23). The mutant P6 Asp residue is cyan. **(B)** Interactions between TCR JDI and wild-type KRAS peptide (7OW5) (23). **(C)** Conformational changes in mutant and wild-type KRAS peptides upon binding TCR JDI. Superposition of unbound KRAS^G12D^–HLA-A*11 and KRAS–HLA-A*11 ligands shows that the peptides adopt an open conformation (7OW4 and 7OW3) (23). Superposition of TCR-bound KRAS^G12D^–HLA-A*11 and KRAS–HLA-A*11 ligands shows that the peptides adopt a closed conformation. **(D)** Interaction network between P6 Asp and HLA F pocket residues in the TCR JDI–KRAS^G12D^–HLA-A*11 complex.

As discussed above, poor presentation of wild-type KRAS by HLA-C*08:02 is likely the mechanism underpinning TCR specificity for KRAS^G12D^ (22). By contrast, the KRAS and KRAS^G12D^ peptides bound equally well to HLA-A*11:01, ruling out TCR selectivity based on peptide presentation. Crystal structures of TCRs JDI and JDIa41b1 bound to KRAS^G12D^–HLA-A*11, and of JDIa41b1 bound to wild-type KRAS–HLA-A*11, have provided insights into the molecular basis for TCR selectivity for KRAS^G12D^ (**Figures 4A, B**) (23). These structures showed no significant differences in TCR interactions with KRAS^G12D^ versus wild-type KRAS. However, structures of KRAS^G12D^–HLA-A*11 and KRAS–HLA-A*11 without a bound TCR revealed that both mutant and wild-type KRAS peptides underwent an induced fit conformational change upon TCR engagement (**Figure 4C**), with P6 Asp in the KRAS^G12D^–HLA-A*11 complex forming multiple stabilizing interactions with the HLA-A*11 F pocket (**Figure 4D**). Thermodynamic analysis and molecular dynamics simulations indicate that tighter TCR binding to KRAS^G12D^–HLA-A*11 compared to KRAS–HLA-A*11 is driven by a greater net formation of new electrostatic interactions with TCR by the mutant peptide, at the cost of a greater order-disorder transition (23). Thus, as in the case of p53^R175H^ recognition by TCR 6-11 (21), TCRs can use indirect energetically driven strategies for preferential neoantigen binding.

### TCR recognition of KRAS^G12V^–HLA-A*11:01

Besides KRAS^G12D^, another frequent oncogenic mutation in KRAS is replacement of glycine at position 12 by valine (KRAS^G12V^) (51). Lu et al. isolated TCRs (1-2C and 3-2E) specific for a KRAS^G12V^ nonamer (VVGA**V**GVGK) by immunizing HLA-A*11:01 transgenic mice with this peptide (**Table 1**) (24). As measured by SPR, TCRs 1-2C and 3-2E bound mutant KRAS^G12V^–HLA-A*11 with *K*_D_s of 14 μM and 28 μM, respectively, compared to 131 μM and 42 μM, respectively, for binding to wild-type KRAS–HLA-A*11.

**Figure 5:**
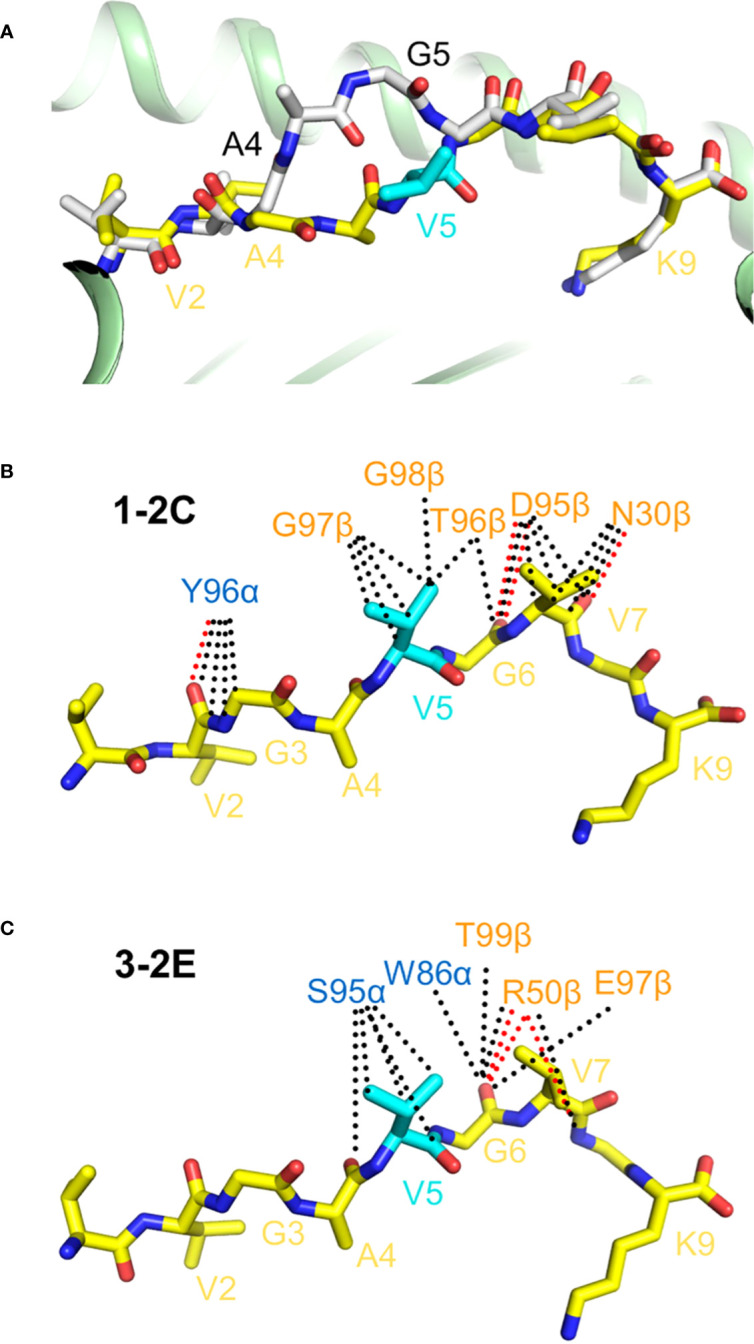
TCR recognition of the HLA-A*11:01-restricted KRAS^G12V^ neoepitope. **(A)** Superposition of wild-type KRAS–HLA-A*11:01 and mutant KRAS^G12V^–HLA-A*11:01 structures (8I5E and 8I5C) (24). Wild-type KRAS peptide is gray; mutant KRAS^G12V^ peptide is yellow. The mutant P5 Val residue is cyan. **(B)** Interactions between TCR 1-C2 and the KRAS^G12V^ peptide. Hydrogen bonds are red dotted lines and van der Waals contacts are black dotted lines. **(C)** Interactions between TCR 3-2E and the KRAS^G12V^ peptide.

Structures of 1-2C and 3-2E bound to KRAS^G12V^–HLA-A*11, and of KRAS–HLA-A*11 in free form, provided insight into how these TCRs discriminate between wild-type and mutant KRAS (24). The glycine-to-valine mutation in the neoepitope is located at P5 at the center of the peptide. Residues P4 and P5 of KRAS^G12V^ are shifted significantly downward towards the peptide-binding groove of HLA-A*11 compared to their positions in wild-type KRAS, thereby avoiding steric clashes with TCRs 1-2C and 3-2E (**Figure 5A**). Moreover, both TCRs target the P5 Val driver mutation, albeit through completely different sets of interactions (**Figures 5B, C**). Therefore, specific TCR recognition of KRAS^G12V^ depends not only on its distinct conformation compared to the wild-type peptide, but also on extensive direct contacts with the mutant P5 Val residue, as observed for TCR recognition of p53^R175H^ (20, 21).

### TCR recognition of HHAT^p8F^–HLA-A*02:06

**Figure 6:**
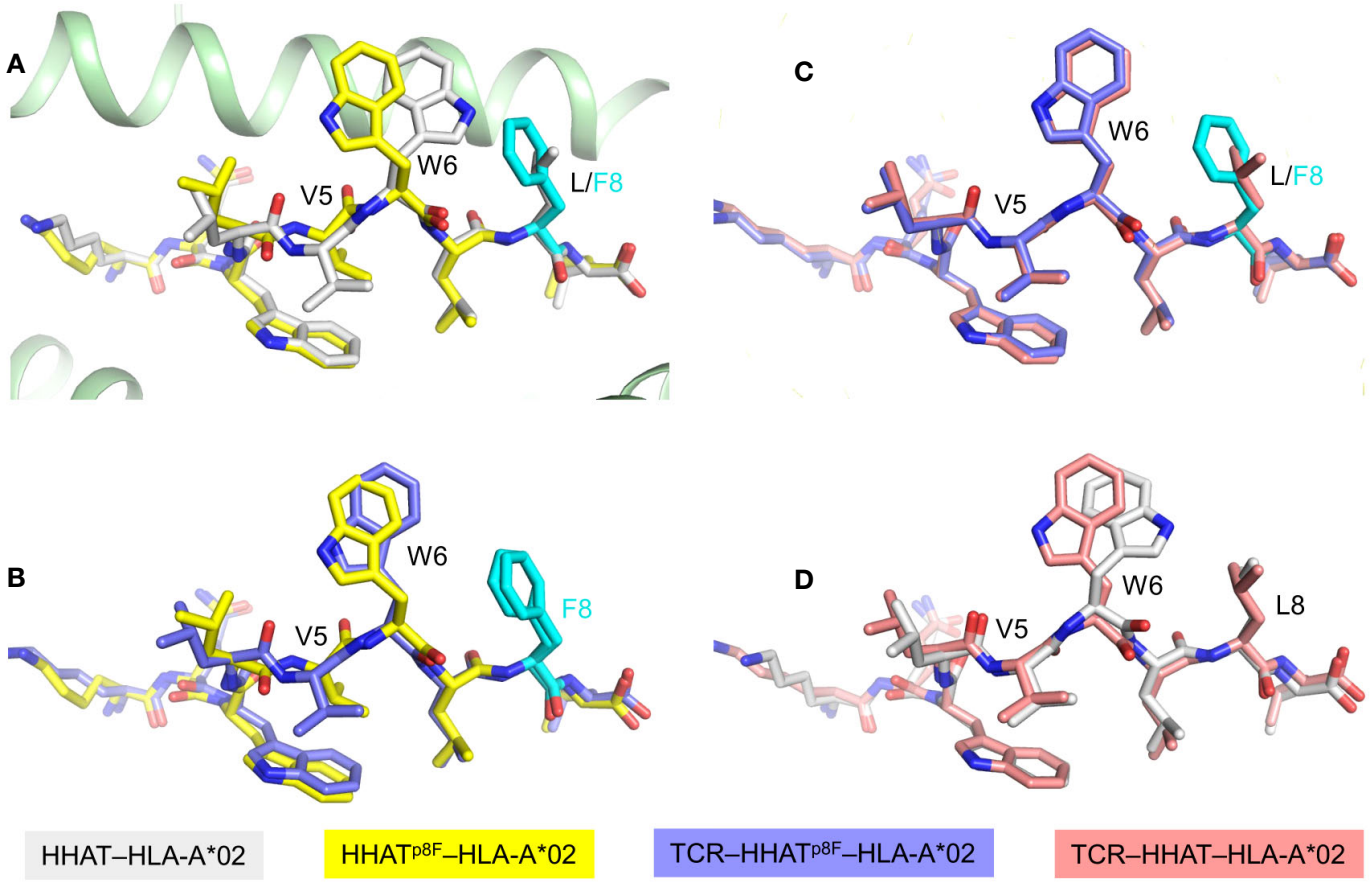
Pre-organization of the HHAT^p8F^ neoepitope by an immunogenic mutation. **(A)** Superposition of unbound wild-type HHAT–HLA-A*02 and mutant HHAT^p8F^–HLA-A*02 structures (6UJQ and 6UJO) (25). The mutant P8 Phe residue is cyan. The conformations of the mutant and wild-type peptides are similar except for the orientation of the P6 Trp side chain. **(B)** Comparison of HHATp8F peptide conformations in unbound versus TCR-bound HHAT^p8F^–HLA-A*02 structures (6UK4) (25). The mutant peptide in the TCR 302TIL–HHAT^p8F^–HLA-A*02 complex adopts the same conformation as in unbound HHAT^p8F^–HLA-A*02. **(C)** Superposition of the peptides in the neoepitope and wild-type TCR–pMHC complexes shows they have identical conformations (6UK2) (25). **(D)** The orientation of the P6 Trp side chain in unbound HHAT–HLA-A*02 differs from that in the TCR 302TIL–HHAT–HLA-A*02 complex.

The HHAT^p8F^ neoepitope was first identified in ovarian cancer patients and derives from the hedgehog acyltransferase (HHAT) oncogene (57). It is restricted by HLA-A*02:06 and incorporates a leucine-to-phenylalanine substitution at P8 (KQWLVWL**F**L). Patient-derived CD8^+^ TILs strongly recognized HHAT^p8F^ but not wild-type HHAT. Although these peptides bound equally well to HLA-A*02:06, as measured by differential scanning fluorimetry, crystal structures of the wild-type HHAT–HLA-A*02 and mutant HHAT^p8F^–HLA-A*02 complexes showed that the peptides differ most in the orientation of the side chain of P6 Trp, which is two residues away from the leucine-to-phenylalanine mutation at P8 (**Figure 6A**) (25). A 120° rotation of the P6 Trp side chain is induced by P8 Phe, which would clash with the P6 Trp side chain if it maintained the conformation seen in wild-type HHAT.

TCR 302TIL, isolated from TILs of an ovarian cancer patient, bound HHAT^p8F^–HLA-A*02 with *K*_D_ = 9 μM compared to 200 μM for HHAT–HLA-A*02 (**Table 1**) (25). This 20-fold affinity differential can be explained by the structure of TCR 302TIL bound to HHAT^p8F^– HLA-A*02. In this complex, the mutant peptide adopts the same conformation seen in unbound HHAT^p8F^–HLA-A*02, with the P6 Trp side chain displaying a nearly identical orientation (**Figure 6B**). Notably, in the structure of TCR 302TIL bound to wild-type HHAT–HLA-A*02, P6 Trp adopts the same configuration as in the neoepitope complex (**Figure 6C**), which differs from that found in unbound HHAT–HLA-A*02 (**Figure 6D**). Therefore, the P8 Phe mutation pre-organizes the P6 Trp side chain into a conformation optimal for recognition by a neoantigen-specific TCR, in this way converting a self-epitope into an immunogenic epitope and enabling T cells to mediate killing of tumor but not normal cells.

### TCR recognition of PIK3CA^H1047L^–HLA-A*03:01

*PIK3CA* encodes phosphoinositide 3-kinase α (PI3Kα), which is involved in cell proliferation, differentiation, motility, and survival, which are key cellular functions in cancer development. *PIK3CA* is among the most common genetically altered driver oncogenes, with the largest portion of mutations occurring at hotspot position H1047 (58, 59). Chandran et al. (26) isolated several TCRs from the T cells of a healthy donor that recognize a neoepitope corresponding to residues 1046–1054 of PIK3CA^H1047L^ which contains a histidine-to-leucine mutation at position 1047 (A**L**HGGWTTK) (**Table 1**). These TCRs are restricted by the prevalent HLA-A*03:01 allele.

**Figure 7:**
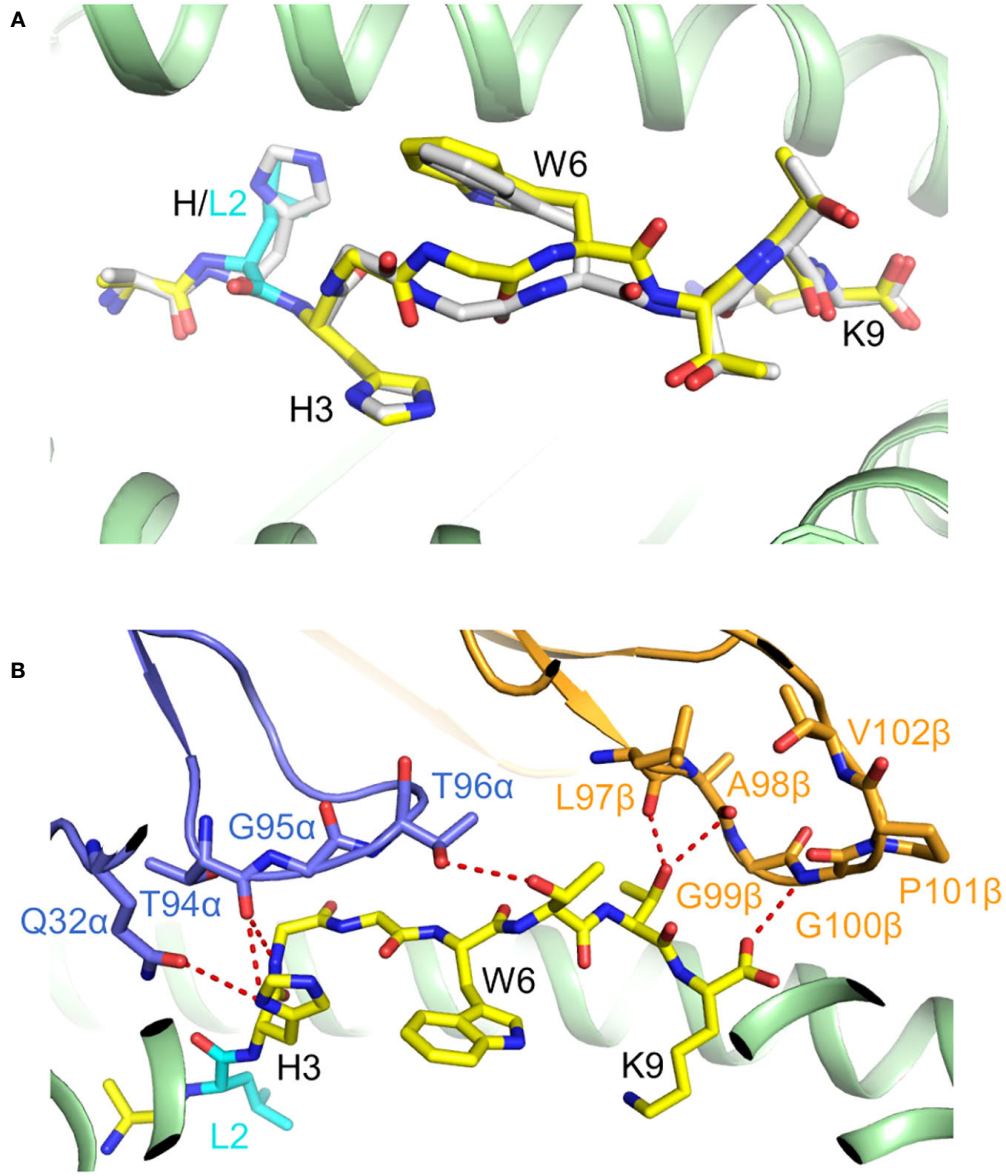
Presentation of PIK3CA^H1047L^ neoepitope by HLA-C*03:01. **(A)** Superposition of mutant PIK3CA^H1047L^–HLA-A*03 and wild-type PIK3CA–HLA-A*03 complexes (7L1B and 7L1C) (26). The conformations of the mutant (yellow) and wild-type (gray) peptides are nearly identical. **(B)** Interactions between TCR4 and the PIK3CA^H1047L^ peptide (7RRG) (26).

Crystal structures of PIK3CA and PIK3CA^H1047L^ peptides bound to HLA-A*03:01 showed that the wild-type and mutant peptides adopt nearly identical conformations, which cannot explain the immunogenic potential of the PIK3CA^H1047L^ neoepitope (**Figure 7A**). However, the thermal stability (melting temperature) of the mutant PIK3CA^H1047L^–HLA-A*03 complex (54 °C) is considerably higher than that of the wild-type PIK3CA–HLA-A*03 complex (37 °C) (26). Moreover, the half-life of the PIK3CA^H1047L^–HLA-A*03 complex (5.50 hours) is ~70 times longer than the half-life of the PIK3CA–HLA-A*03 complex (0.08 hours). The greater stability of the neoepitope complex is attributable to an optimal anchor residue at P2 (leucine rather than histidine) as a consequence of the mutation. As in the case of KRAS^G12D^ (22), the immunogenicity of PIK3CA^H1047L^ arises from preferential presentation by MHC class I rather than differential TCR recognition.

The structure of a PIK3CA^H1047L^-specific TCR (TCR4) bound to PIK3CA^H1047L^–HLA-A*03 revealed that TCR4 possesses an unusually long CDR3β loop that enables this TCR to form an extended and highly complementary interface with the neoepitope (**Figure 7B**). Importantly, adoptive transfer of TCR4-transduced T cells led to tumor regression in mice bearing mutant PIK3CA^H1047L^ tumors but not wild-type PIK3CA tumors, which supports the clinical potential of TCR4 for ACT (26).

### Recognition of cancer neoantigens by antibody mimics of TCRs

In addition to TCRs, monoclonal antibodies are also under investigation for immunotherapeutic targeting of cancer neoantigens (30, 60). These antibodies, called TCR-mimic antibodies (TCRm Abs), are designed to recognize pMHC complexes on cancer cell surfaces, similar to TCRs. They are typically isolated by screening phage or yeast libraries displaying single-chain Fv fragments with recombinant forms of the target pMHC. TCRm Abs have higher affinity than TCRs and can be readily converted to therapeutic formats such as bispecific antibodies, antibody–drug conjugates, and chimeric antigen receptors (CARs).

TCRm Abs have been described that specifically recognize several cancer neoantigen–HLA complexes, including p53^R175H^–HLA-A2 (61), KRAS^G12V^–HLA-A3 (62, 63), RAS^Q61H^–HLA-A1 (62), IDH2^R140Q^–HLA-B7 (64), and phosphoIRS2–HLA-A2 (65). Structural information is available for how TCRm Abs recognize three of these neoantigen–HLA complexes (**Table 2**) (61, 63, 64). As described below, these TCRm Abs dock on pMHC in ways that are very different from the canonical diagonal orientation of *bona fide* TCRs, in which Vα is positioned over the α2 helix of MHC class I and Vβ over the α1 helix. In the case of TCRs, this highly conserved docking mode is mandated by T cell signaling constraints that optimally localize CD8/Lck to CD3 in the TCR–CD3 complex (66). TCR mAbs are not subject to such signaling constraints because they are simply selected for their ability to bind pMHC targets (30, 60). This allows them to employ diverse strategies to achieve specific recognition.

**Table 2:** Structures of TCR-mimic (TCRm) antibodies bound to cancer neoantigen pMHC ligands.

TCRm–pMHC complex	PDB code (reference)	Neoepitope	Affinity wild-type	Affinity mutant	Basis for neoepitope specificity
H2–p53^R175H^–HLA-A*02:01	6W51 (61)	HMTEVVR**H**C	UD	86 nM	Direct antibody contacts with mutation
V2–KRAS^G12V^–HLA-A*03:01	7STF (63)	VVVGA**V**GVGK	UD	24 nM	Direct antibody contacts with mutation, peptide induced fit
2Q1–IDH2^R140Q^–HLA-B*07:02	6UJ9 (64)	SPNGTI**Q**NIL	846 nM	44 nM	Limited direct antibody contact with mutation, engagement of mutant peptide backbone conformation

UD, undetectable.

TCRm Ab H2 recognizes p53^R175H^–HLA-A2 (61), the exact same pMHC targeted by TCRs described above (20, 21). H2 bound p53^R175H^–HLA-A2 with nanomolar affinity but showed no detectable binding to wild-type p53–HLA-A2, thereby mimicking the exquisite neoepitope specificity of TCRs. H2 was converted to a T cell-based immunotherapeutic by fusing it to an anti-CD3 antibody (61). This bispecific antibody effectively activated T cells to lyse cancer cells presenting the p53^R175H^ neoantigen both *in vitro* and in mice, despite the low density of p53^R175H^–HLA-A2 complexes on the cancer cell surface.

**Figure 8:**
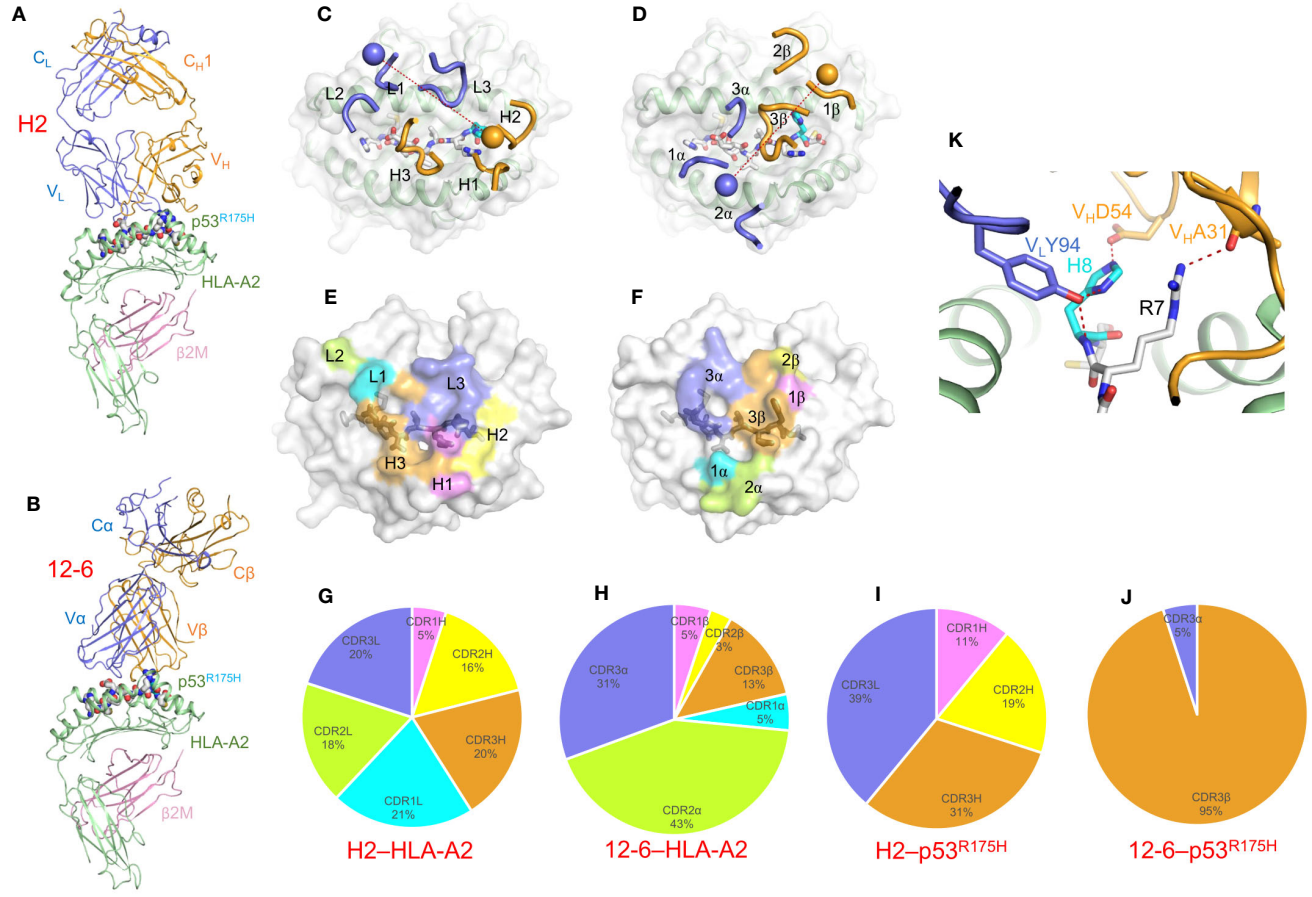
Recognition of p53^R175H^–HLA-A2 by a TCR-mimic antibody. **(A)** Side view of the TCRm Ab H2–p53^R175H^–HLA-A2 complex (6W51) (61). **(B)** Side view of the TCR 12-6–p53^R175H^–HLA-A2 complex (6VRM) (20). **(C)** Positions of CDR loops of TCRm Ab H2 on p53^R175H^–HLA-A2. V_L_CDRs of H2 are shown as numbered blue (L1, L2, and L3) loops; V_H_CDRs are shown as numbered gold (H1, H2, and H3) loops. The p53^R175H^ peptide is drawn in gray in stick representation with the mutant P8 His residue in cyan. HLA-A2 is depicted as a light gray surface. The blue and gold spheres mark the positions of the conserved intrachain disulfide of V_L_ and V_H_, respectively. The red dashed line indicates the crossing angle of TCRm Ab to pMHC. **(D)** Positions of CDR loops of TCR 12-6 on p53^R175H^–HLA-A2. CDRs of 12-6 are shown as numbered blue (CDR1α, CDR2α, and CDR3α) or gold (CDR1β, CDR2β, and CDR3β) loops. The blue and gold spheres mark the positions of the conserved intrachain disulfide of Vα and Vβ, respectively. The red dashed line indicates the crossing angle of TCR to pMHC. **(E)** Footprint of TCRm Ab H2 on p53^R175H^–HLA-A2. The areas contacted by individual CDR loops are color-coded. **(F)** Footprint of TCR 12-6 on p53^R175H^–HLA-A2. **(G)** Pie chart showing percentage distribution of TCRm Ab H2 contacts to HLA-A2 according to CDR. **(H)** Pie chart showing percentage distribution of TCR 12-6 contacts to HLA-A2 according to CDR. **(I)** Pie chart showing percentage distribution of TCRm Ab H2 contacts to p53^R175H^ peptide according to CDR. **(J)** Pie chart showing percentage distribution of TCR 12-6 contacts to p53^R175H^ peptide according to CDR. **(K)** Close-up of interactions between TCRm Ab H2 and P8 His.

In the structure of TCRm Ab H2 bound to p53^R175H^–HLA-A2 (**Figure 8A**) (61), V_L_ (analogous to Vα) is positioned mainly over the α1 helix of HLA-A2 and V_H_ (analogous to Vβ) over the α2 helix (**Figure 8C**), which is nearly the reversed docking polarity of TCR 12-6 on p53^R175H^–HLA-A2 (**Figures 8B, D**). As a consequence, H2 and 12-6 make very different footprints on the pMHC surface (**Figures 8E, F**). The contributions made by individual CDRs to interactions with MHC (**Figure 8G, H**) and peptide (**Figures 8I, J**) differ radically. In the TCRm Ab H2–p53^R175H^–HLA-A2 complex (61), the V_L_CDR3 and V_H_CDR1–3 loops form a tight cage enveloping P7 Arg and P8 His as part of a hydrogen bonding network with V_L_CDR3 Tyr94 and V_H_CDR2 Asp54 (**Figure 8K**). Therefore, H2, like TCR 12-6 (**Figure 2F**), distinguishes mutant from wild-type p53 through direct contacts with P8 His, which is fundamentally different from the indirect strategy employed by TCR 6-11 (21).

**Figure 9:**
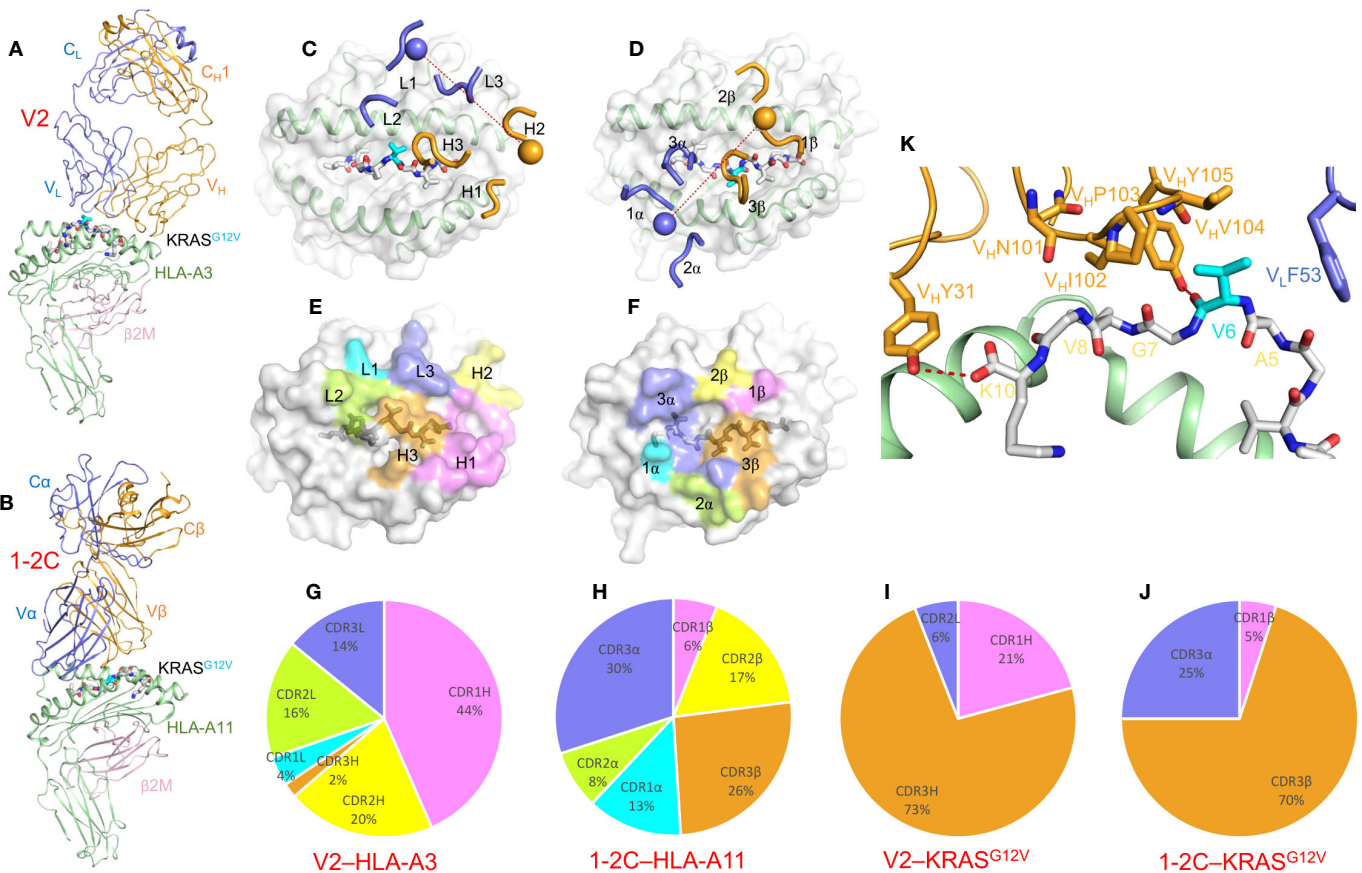
Recognition of KRAS^G12V^–HLA-A*03:01 by a TCR-mimic antibody. **(A)** Side view of the TCRm Ab V2–KRAS^G12V^–HLA-A*03:01 complex (7STF) (63). **(B)** Side view of the TCR 1-2C–KRAS^G12V^–HLA-A*11:01 complex (8I5C) (24). **(C)** Positions of CDR loops of TCRm Ab V2 on KRAS^G12V^–HLA-A*03:01. V_L_CDRs of V2 are shown as numbered blue (L1, L2, and L3) loops; V_H_CDRs are shown as numbered gold (H1, H2, and H3) loops. The KRAS^G12V^ peptide is drawn in gray in stick representation with the mutant P6 Val residue in cyan. HLA-A3 is depicted as a light gray surface. The blue and gold spheres mark the positions of the conserved intrachain disulfide of V_L_ and V_H_, respectively. The red dashed line indicates the crossing angle of TCRm Ab to pMHC. **(D)** Positions of CDR loops of TCR 1-2C on KRAS^G12V^–HLA-A*11:01. CDRs of 1-2C are shown as numbered blue (CDR1α, CDR2α, and CDR3α) or gold (CDR1β, CDR2β, and CDR3β) loops. The blue and gold spheres mark the positions of the conserved intrachain disulfide of Vα and Vβ, respectively. The red dashed line indicates the crossing angle of TCR to pMHC. **(E)** Footprint of TCRm Ab V2 on KRAS^G12V^–HLA-A*03:01. The areas contacted by individual CDR loops are color-coded. **(F)** Footprint of TCR 1-2C on KRAS^G12V^–HLA-A*11:01. **(G)** Pie chart showing percentage distribution of TCRm Ab V2 contacts to HLA-A*03:01 according to CDR. **(H)** Pie chart showing percentage distribution of TCR 1-2C contacts to HLA-A*11:01 according to CDR. **(I)** Pie chart showing percentage distribution of TCRm Ab V2 contacts to KRAS^G12V^ peptide according to CDR. **(J)** Pie chart showing percentage distribution of TCR 1-2C contacts to KRAS^G12V^ peptide according to CDR. **(K)** Interactions between TCRm Ab V2 and the KRAS^G12V^ peptide.

Another TCRm Ab, V2, specifically recognizes KRAS^G12V^ presented by HLA-A*03:01 (62, 63), which is closely related to the pMHC targeted by TCR 1-2C (KRAS^G12V^–HLA-A*11:01) (24). A bispecific antibody constructed by fusing V2 to an anti-CD3 antibody induced T cell activation and killing of target cancer cells expressing endogenous levels of KRAS^G12V^ neoantigen (62). In the structure of TCRm Ab V2 bound to KRAS^G12V^–HLA-A*03 (**Figure 9A**) (63), V_L_ and V_H_ are both positioned over the α1 helix of HLA-A*03 (**Figure 9C**), in sharp contrast to the canonical docking topology of TCR 1-2C (**Figures 9B, D**). As a result, the footprints of V2 and 1-2C on the pMHC surface are very different (**Figures 9E, F**), as are the contributions made by individual CDRs to interactions with MHC (**Figures 9G, H**). However, V_H_CDR3 of V2, like CDR3β of 1-2C, dominates contacts with the KRAS^G12V^ peptide (**Figures 9I, J**). V2 engages the mutation site at P5 Val with a loose hydrophobic cage comprising V_L_Phe53, V_H_Pro103, V_H_Val104, and V_H_Tyr105, with the N-terminal portion of the KRAS^G12V^ peptide nearly completely untouched by V2 (**Figure 9K**) (63).

TCRm Ab 2Q1 specifically recognizes a neoantigen derived from isocitrate dehydrogenase 2 (IDH2^R140Q^) (SPNGTI**Q**NIL) presented by HLA-B*07:02 (64). CAR T cells constructed from 2Q1 were cytotoxic against IDH2^R140Q^-bearing target cells. Structures of wild-type IDH2–HLA-B*02 and mutant IDH2^R140Q^–HLA-B*02 complexes showed that the peptides bound in essentially identical conformations and that P7 Arg/Gln (wild-type and mutant amino acids) is buried deep within the peptide-binding groove of the MHC molecule (64). In the structure of TCRm Ab 2Q1 bound to IDH2^R140Q^–HLA-B*02, the only direct interaction between P7 Gln and 2Q1 is a hydrogen bond linking P7 Gln to V_H_CDR3 Arg102. Elimination of this hydrogen bond by mutating V_H_CDR3 Arg102 to alanine completely abrogated TCRm Ab binding (64). 2Q1 docks onto pMHC in a parallel orientation rather than the canonical diagonal orientation of *bona fide* TCRs.

Similar to neoantigen-specific TCRm Abs (61, 63, 64), TCRm Abs specific for unmutated tumor-associated antigens such as MAGE-A1 and ESK1 also do not need to mimic TCRs completely to achieve peptide-specific recognition (67, 68). For example, a TCRm Ab specific for MAGE-A1–HLA-A1 focused on the HLA-A1 α1 helix with no contacts to N-terminal peptide residues (67). Another TCRm Ab specific for MART-1–HLA-A1 engaged pMHC with a TCR-like docking angle but its V_H_CDR1 and V_H_CDR2 loops were completely absent from MHC interactions (30). Collectively, structural studies of TCRm Ab–pMHC complexes have revealed a wide range of docking orientations that can diverge substantially from the canonical diagonal orientation of natural TCR–pMHC complexes without compromising specificity.

### Predicting neoepitope immunogenicity

Accurate computational prediction of immunogenic neoantigens is of high interest for neoantigen-based vaccines and therapeutics. Neoepitope prediction faces a number of challenges, including 1) predicting whether peptides containing the somatic mutation of interest are actually generated during antigen processing, 2) predicting whether these peptides come into contact with MHC molecules in the MHC class I or class II antigen presentation pathway, 3) predicting which peptides bind MHC with sufficient affinity to be presented on the cell surface, and 4) predicting whether the displayed neoepitope–MHC complexes can be recognized by TCRs. Although much progress has been made towards meeting these challenges, at least for MHC class I-restricted neoepitopes, considerable obstacles remain.

Several recent studies have demonstrated the utility of structural information to generate neoepitope predictions or to provide key insights into predictions. In a 2019 study, Riley et al. performed modeling of candidate peptides in complex with HLA-A2, and utilized energetic features from those structural models to train a neural network predictor (69). Their method performed well relative to other methods based on their benchmarking, and while only predicting immunogenicity of nonameric peptides presented by HLA-A2 limited its practical applicability, it provided a proof of concept of such a structure-based approach, as the authors noted. A recent study that characterized neoantigen-specific TCR structural avidity utilized structural modeling in that context, finding that the amount of predicted interface contacts in template-based models of TCR–pMHC complexes was associated with higher avidity (70). Additionally, the authors generated a structure-based logistic regression model to predict avidity of TCRs without the antigen context.

Other computational neoepitope prediction studies have utilized structures to interpret and contextualize their results. One method named PRIME can predict MHC class I T cell neoepitopes based on MHC binding affinity and TCR recognition propensity (71); that algorithm performed favorably against other prediction tools, and an updated version of that algorithm (PRIME2.0) has been reported (72). In the original PRIME study, the authors found that the trained algorithm, which did not explicitly take structural information into account, was in agreement with structural features of individual TCR–pMHC complex interfaces as well as overall interface residue preferences in TCR–pMHC complex structures (71). The authors of the recently reported deep learning neoepitope prediction algorithm BigMHC reported improved performance over PRIME2.0 and a number of other predictive methods in neoepitope immunogenicity prediction (73), and they mapped the deep learning attention encodings (trained, as with the PRIME method, on sequence data) onto MHC structures to gain insights into key MHC residues.

While relatively few methods to date have directly utilized structural information in neoepitope immunogenicity prediction, with PRIME, BigMHC, and other recent machine learning-based methods (74–76) using sequence data for training, the modeling approaches noted above suggest that structural data may be helpful in prospective algorithm developments. With recent advances in deep learning-based structural modeling including AlphaFold (77), and adaptations of AlphaFold and related deep learning methods to accurately model TCRs (78), pMHCs (79), and TCR–pMHC complexes (80, 81), it is likely that such approaches would be helpful in that context. Currently determined structures of neoepitope-containing TCR–pMHC complexes (**Table 1**) help to illustrate the structural basis of concepts such as “agretopicity”, which is preferential mutant epitope binding by MHC (82) and is considered directly in predictive computational methods (e.g. 74, 76); additional structures of these complexes may provide new neoepitope recognition features that in turn can inform future method developments.

## Conclusions

Structural studies have revealed that cancer neoepitopes need differ only slightly from their wild-type counterparts for them to be immunogenic in patients. However, this physiochemical similarity presents a challenge to the immune system and probably explains, at least in part, the low frequency of T cells able to recognize neoantigens with sufficient avidity to mediate efficient killing of tumor cells. Some neoepitope mutations increase peptide–MHC binding, thereby improving antigen presentation (e.g. KRAS^G12D^ and PIK3CA^H1047L^). Other mutations increase affinity for TCR, either through direct contacts with TCR (e.g. TPI^T28I^, p53^R175H^, and KRAS^G12V^) or via indirect mechanisms such as conformational pre-organization of pMHC (e.g. HHAT^p8F^). TCRm Abs use non-canonical docking topologies to recognize pMHC and provide an alternative to TCRs for immunotherapeutic targeting of cancer neoantigens. Structure-guided engineering of TCRs (23) and TCRm Abs (30, 64) provides a means for optimizing these molecules for ACT, including with CAR T cells, as well as for incorporating TCRs and TCRm Abs into alternative therapeutic formats such as bispecific agents and drug conjugates.

## Author contributions

RM: Writing – original draft, Writing – review & editing. DW: Writing – original draft, Writing – review & editing. BP: Writing – original draft, Writing – review & editing.
